# A Case Report of Olmesartan-Induced Enteropathy

**DOI:** 10.7759/cureus.20722

**Published:** 2021-12-26

**Authors:** Benjamin S Daines, Alfred Kankam Jr., Sadia Tanami, Rajesh Nambiar

**Affiliations:** 1 Internal Medicine, Texas Tech University Health Sciences Center, Amarillo, USA

**Keywords:** olmesartan-induced enteropathy, celiac disease, diarrhea, enteropathy, olmesartan

## Abstract

Chronic olmesartan use can cause a drug-induced enteropathy as a rare side effect leading to diarrhea, significant weight loss, and reduced quality of life. The mechanism of this enteropathy is poorly understood and requires further investigation. We present a case of olmesartan-induced enteropathy resulting in recurrent hospitalizations for intractable diarrhea. Significant enteropathy is more commonly related to infectious or autoimmune causes making the diagnosis of drug-induced enteropathy a challenge. In this case, the lack of significant findings on labs or imaging resulted in a thorough diagnostic work-up revealing olmesartan-induced enteropathy. We present this case to inform providers of the possibility of olmesartan-induced enteropathy and characteristics to identify in other similar cases.

## Introduction

Olmesartan is an antihypertensive angiotensin-receptor blocker (ARB) originally approved by the U.S. Food and Drug Administration (FDA) in 2002 [1]. Olmesartan is a popular antihypertensive drug due to its specificity for type 1 angiotensin II receptors, lack of demonstrated pharmacokinetic interaction with digoxin and warfarin, and greater lowering of seated diastolic blood pressure compared to losartan, valsartan, and captopril [2]. Despite the clinical benefits of olmesartan, sprue-like enteropathy was reported as a side effect of chronic olmesartan use resulting in significant diarrhea and weight loss [3]. In patients reporting this side effect, cessation of the drug can result in rapid improvement of symptoms [3]. Diagnosis requires ruling out other causes of enteropathy including celiac disease, infectious gastroenteritis, and drug rash with eosinophilia and systemic symptoms (DRESS) syndrome presenting with colitis [4]. Increased awareness of olmesartan as a potential cause of drug-induced enteropathy can reduce unnecessary testing and invasive procedures.

## Case presentation

A 54-year-old male with a past medical history of hypertension, type II diabetes mellitus, and gout presented to the emergency department (ED) with a chief complaint of watery diarrhea for the past month. He denied bloody or mucoid stools. He said diarrhea occurred over 10 times per day, occasionally woke him up at night, and is unrelated to oral consumption. Over the previous month, he endorsed unintentional 45-pound weight loss, nausea, chills, reduced oral intake, fatigue, and intermittent fever. He denied vomiting, abdominal pain, and fatty or foul-smelling stools. He denied recent travel and endorsed a monogamous relationship with his wife. The patient was previously admitted to the hospital two weeks earlier with the same presentation, was stabilized with supportive care, and was discharged after three days. The previous work-up included negative stool pathogen panel, negative stool ova, and parasite test, negative Clostridium difficile toxin assay, and normal colonoscopy with unremarkable intestinal biopsies.

Initial evaluation revealed stable vitals and admission laboratory findings in Table 1. Abdominal CT was unrevealing for inflammatory bowel disease or intra-abdominal abnormalities. No identifiable infectious etiology led to additional testing for other causes of intractable watery diarrhea including amyloidosis, carcinoid syndrome, celiac disease, intestinal tuberculosis, VIPoma, and Zollinger-Ellison syndrome. Follow-up labs revealed normal urine protein electrophoresis and fixation, negative tissue transglutaminase antibodies, negative interferon-gamma release assay, and normal gastrin levels. Chromogranin A levels were elevated at 542 ng/mL (normal range: < 311 ng/ml) but 24-hour urine 5-hydroxyindoleacetic acid (5-HIAA) returned normal at 6.4 mg/24hr (normal range: 2-9 mg/24hr). These findings indicated a different etiology of the enteropathy, leading to medication reconciliation. For the past three years, the patient was taking olmesartan, an ARB associated with sprue-like enteropathy. With no identifiable trigger, olmesartan was discontinued to rule out olmesartan-induced enteropathy. The patient was transitioned to IV hydralazine for blood pressure control with IV fluids and clear liquids for electrolyte correction. He experienced moderate improvement of diarrheal symptoms and was discharged after three days.

**Table 1:** Laboratory Results WBC: white blood cells.

Investigation	Value	Reference Range
WBC (K/µL)	7.2	4.8-10.8
Hemoglobin (g/dL)	13.0	14.0-18.0
Platelets (K/µL)	336	130-400
Sodium (mmol/L)	129	135-145
Potassium (mmol/L)	4.7	3.5-5.0
Chloride (mmol/L)	109	98-107
Bicarbonate (mmol/L)	11	21-31
C-Reactive Protein (mg/dL)	0.880	<1.000
Erythrocyte Sedimentation Rate (mm/hr)	24	0-20
Procalcitonin (ng/mL)	0.10	<0.05

The patient presented to the ED three days later for sharp chest pain on inspiration due to a pericardial effusion. At this time, he endorsed the complete resolution of his diarrheal symptoms stating he was now having one solid stool per day. He experienced no relapse of his enteropathy during this four-day hospital stay where his pericardial effusion resolved with supportive care. At his cardiology follow-up two weeks later, the patient continued to endorse normal stooling once per day and his first weight gain in over six weeks. The patient was advised to return for any relapse in symptoms.

## Discussion

Enteropathy with significant diarrhea and weight loss produces a broad differential with autoimmune causes including celiac disease, infectious causes including tropical sprue, inflammatory causes including microscopic colitis, malignant causes including carcinoid syndrome, infiltrative causes including amyloidosis, and drug-induced causes including olmesartan and DRESS syndrome [4-5]. The variety of etiologies poses a diagnostic challenge for physicians, especially in the case of drug-induced enteropathy where common diagnostic procedures such as stool pathogen panels and colonoscopy may be unrevealing. Exclusion of more common causes of enteropathy such as celiac disease must be done given the similar clinical presentation of olmesartan-induced enteropathy with small intestinal villous blunting and inflammatory cell infiltration [6]. Differentiation can be made by testing for celiac serology. Despite potential similarities in presentation, olmesartan-induced enteropathy demonstrates impressive histologic variability. This indicates the need to attempt a trial of olmesartan cessation in patients taking the drug presenting with enteropathy of unknown etiology.

In this case, attempts were initially made to relate the pericardial effusion to the patient's significant enteropathy. Multiple potential causes including carcinoid syndrome, intestinal tuberculosis, amyloidosis, and Whipple disease were suspected and subsequently ruled out with normal 5-HIAA, negative interferon-gamma release assay, normal protein electrophoresis, and fixation, and negative intestinal biopsy respectively [4]. The presentation of the pericardial effusion nearly a week after olmesartan cessation was puzzling, but the resolution of pericardial effusion with supportive care and lack of recurrent symptoms at cardiology follow-up indicated a likely idiopathic cause [7].

The mechanism of olmesartan-induced enteropathy is poorly understood and requires greater investigative efforts. The delay between the initiation of olmesartan and symptoms of enteropathy reduces the likelihood of a type I hypersensitivity reaction [8]. Previous research has found the average time between drug initiation and symptom onset to be 3.1 years [3]. Increased levels of angiotensin II due to ARBs have been shown to induce transforming growth factor beta (TGF-beta) expression, which mediates various intestinal epithelial inflammatory factors [9-10]. While this pathway may explain the mechanism of olmesartan-induced enteropathy, it does not explain the lack of enteropathy-like symptoms with other ARBs. Replacement of olmesartan in cases of drug-induced enteropathy with losartan has led to symptom resolution indicating an olmesartan-specific mechanism [6]. Previous case reports have demonstrated both symptomatic and histologic improvement in the majority of patients following drug cessation [3]. Some patients have demonstrated complete resolution of symptoms [6]. While drug cessation often leads to symptomatic improvement, resistant cases are possible which may benefit from steroids [11].

Olmesartan is a generally well-tolerated ARB with dizziness as the most commonly reported side effect [12]. Few adverse effects were reported until 2012 when sprue-like enteropathy was originally reported as a potential side effect [3]. Multiple subsequent case reports have corroborated these side effects and demonstrated the variability in both subjective and objective findings of olmesartan-induced enteropathy. This variability is evident in the case we report, where the patient demonstrated no histopathologic abnormalities on colonoscopy. This is unusual, given initial case reports demonstrated a majority of patients with villous atrophy, active inflammation, or intraepithelial lymphocytes [3]. The difficulty in establishing a diagnosis of olmesartan-induced enteropathy can lead to treatment delays, unnecessary use of hospital resources, and worsening patient quality of life. Olmesartan-induced enteropathy should be an important consideration on a differential for any patient taking the drug presenting with unexplained enteropathy.

## Conclusions

Olmesartan-induced enteropathy is a rare side effect that can result in a reduction in patient quality of life. The great variability in presentation and histological findings poses a significant diagnostic challenge. We present a case of a 54-year-old man with olmesartan-induced enteropathy lacking typical histologic changes to further elucidate the variability in presentation. Early identification of the side effect of olmesartan cessation can lead to rapid improvement of symptoms while reducing healthcare waste. Future research will be needed to investigate the mechanism of olmesartan-induced enteropathy to better guide antihypertensive therapy.

## References

[1] Chobanian AV, Bakris GL, Black HR (2003). Seventh report of the Joint National Committee on Prevention, Detection, Evaluation, and Treatment of high blood pressure. Hypertension.

[2] Warner GT, Jarvis B (2002). Olmesartan medoxomil. Drugs.

[3] Rubio-Tapia A, Herman ML, Ludvigsson JF, Kelly DG, Mangan TF, Wu TT, Murray JA (2012). Severe spruelike enteropathy associated with olmesartan. Mayo Clin Proc.

[4] Jevtic D, Dumic I, Nordin T (2021). Less known gastrointestinal manifestations of drug reaction with eosinophilia and systemic symptoms (DRESS) syndrome: a systematic review of the literature. J Clin Med.

[5] Camilleri M, Sellin JH, Barrett KE (2017). Pathophysiology, evaluation, and management of chronic watery diarrhea. Gastroenterology.

[6] Adike A, Corral J, Rybnicek D, Sussman D, Shah S, Quigley E (2016). Olmesartan-induced enteropathy. Methodist Debakey Cardiovasc J.

[7] Sagristà-Sauleda J, Mercé AS, Soler-Soler J (2011). Diagnosis and management of pericardial effusion. World J Cardiol.

[8] Dreifuss SE, Tomizawa Y, Farber NJ, Davison JM, Sohnen AE (2013). Spruelike enteropathy associated with olmesartan: an unusual case of severe diarrhea. Case Rep Gastrointest Med.

[9] Takai S, Jin D, Miyazaki M (2010). New approaches to blockade of the renin-angiotensin-aldosterone system: chymase as an important target to prevent organ damage. J Pharmacol Sci.

[10] Feagins LA (2010). Role of transforming growth factor-β in inflammatory bowel disease and colitis-associated colon cancer. Inflamm Bowel Dis.

[11] Hujoel IA, Rubio-Tapia A (2016). Sprue-like enteropathy associated with olmesartan: a new kid on the enteropathy block. GE Port J Gastroenterol.

[12] Scott LJ, McCormack PL (2008). Olmesartan medoxomil: a review of its use in the management of hypertension. Drugs.

